# Chromosomal Heterogeneity of the G-401 Rhabdoid Tumor Cell Line: Unusual Partial 7p Trisomy

**DOI:** 10.3389/fmed.2019.00187

**Published:** 2019-08-30

**Authors:** Elizaveta Fasler-Kan, Nijas Aliu, Frank-Martin Haecker, Natalia Maltsev, Sabrina Ruggiero, Dietmar Cholewa, Andreas Bartenstein, Milan Milošević, Steffen M. Berger

**Affiliations:** ^1^Department of Pediatric Surgery, Children's Hospital, Inselspital, University of Bern, Bern, Switzerland; ^2^Department of Biomedical Research, University of Bern, Bern, Switzerland; ^3^Department of Biomedicine, University of Basel, University Hospital Basel, Basel, Switzerland; ^4^Department of Human Genetics, University Children's Hospital, Inselspital, Bern, Switzerland; ^5^Department of Pediatric Surgery, Children's Hospital of Eastern Switzerland, St. Gallen, Switzerland; ^6^Faculty of Medicine, University of Basel, Basel, Switzerland; ^7^Department of Human Genetics and USA Computation Institute, University of Chicago, Chicago, IL, United States

**Keywords:** chromosomal aberration, partial 7p trisomy, pathophysiology, rhabdoid tumor, FISH assay, proliferation, G-401 cell line

## Abstract

Rhabdoid tumor is a very aggressive and hardly curable pediatric malignancy. It commonly starts in the kidneys but also can occur in the brain, liver, and other organs. The treatment of this tumor usually involves a combination of surgery, radiation, and chemotherapy. Because this tumor is rare, there is still limited experience with a defined standard of care. Cytogenetic analysis is an important routine method to monitor chromosomal aberrations. We have analyzed metaphases of the G-401 rhabdoid tumor cell line. In these cells we have observed metaphases with derivative chromosome 12 arising from partial trisomy 7p. With increasing passage number the numbers of metaphases having this derivative chromosome 12 were found to be higher. In passage number 2 only one metaphase had this pathological chromosome 12. By passage number 10 and passage number 15 about 25 and 95% of this derivative chromosome 12 were found, respectively. We were able to subclone G-401 cells by limiting dilutions and successfully separated cells having apparently normal karyotypes from cells having derivative chromosome 12. Using the cell proliferation assay we showed that clones possessing the derivative chromosome 12 grew more rapidly than clones with normal chromosomes. The cell cycle analysis confirmed this observation. Overall, in this study we describe for the first time a 7p triplication in a rare rhabdoid tumor of kidney. Both types of clones described in this study could be used as a preclinical model to study the involvement of partial chromosome 7 alterations in the development of rhabdoid tumors.

## Introduction

Several types of rare cancers occur predominantly in children and young adults. Rhabdoid tumors of the kidney are highly malignant neoplasms that most commonly occur within the first 3 years of childhood. The median age is 11 months and the male:female ratio 1.5:1. Despite improved knowledge and an increasing number of current methodologies, for many rare cancers the identification of causes or development of strategies for prevention and/or early detection is extremely challenging. Although rhabdoid tumors of the kidney are generally regarded as distinct from Wilms tumor, their pathogenesis remains unclear. The treatment of malignant rhabdoid tumor usually involves a combination of therapies including surgery, radiation and chemotherapy. Rhabdoid tumors have been considered highly malignant with a poor prognosis (1–4).

Within the last 2–3 decades, a significant number of studies confirmed the relevance of genetic abnormalities for diagnosis, therapeutic strategies and prognosis of patients.

Chromosomal aberrations have been well-described in many tumors and tumor cell lines. It is known that chromosomal heterogeneity can drive critical events such as growth and survival advantages, progression and karyotype evolution (5–7).

We have analyzed the chromosomes of the G-401 cell line. This cell line was initially classified as Wilms tumor. However, many researchers believe that this cell line is of rhabdoid tumor origin (8). Cytogenetic studies of rhabdoid tumors have revealed normal karyotypes, a few reports have indicated abnormalities in chromosome regions 22q, 11p13, and 11p15.5 (9–15). In particular chromosomal band 11p15.5. was proven to be an intriguing region of the human genome, since it bears alterations in a variety of tumors (9). Studies of Wilms tumor and rhabdoid tumors support the data regarding tumor suppressor properties of WT2 gene located on 11p15.5 (16–18).

The G-401 cell line was established from a 3-month old infant (8). In this cell line we have observed some cells with an aberrant chromosome 12. With increasing passage numbers more cells with a derivative chromosome 12 have been observed. The FISH analysis showed that these cells carried an additional 7p-segment at the end of the one of chromosomes 12q (partial trisomy 7p). In all cells both chromosomes 7 were structurally normal.

We have subcloned the G-401 by limiting dilutions and successfully separated cells carrying derivative chromosome 12 from the cells having a normal karyotype. After second subcloning every third passage has been karyotyped, in total we have analyzed 33 passages. All clones kept their cytogenetic features. Using the cell proliferation assay we demonstrated that clones possessing the derivative chromosome 12 grew more rapidly than clones with apparently normal chromosomes. The cell cycle data demonstrated that in cells with the 7p translocation the G0/G1 phase comprised 75% and G2/M phase comprised 8%, in contrast in cells which display normal karyotype the G0/G1 and G2/M phase comprised 85 and 6%, respectively.

The DNA profiles of these cells were otherwise identical to those of normal karyotype and from ATCC, therefore excluding contamination by other cell cultures. Since the clones with derivative chromosome 12 have three copies of the 7p-region we propose that the presence of third gene alleles of 7p could have provided growth advantage or that genes located in this locus could regulate the activation of other genes. We have identified 20 genes which could be responsible for this faster proliferation of clones having the 7p translocation and they are a potential target for application of anti-proliferative drugs.

The clones with normal chromosomes and clones with derivative chromosome 12 described in this study might be useful to further study chromosome 7 alterations and the development of this pediatric tumor.

## Materials and Methods

### Cells

The rhabdoid tumor cell line G-401 was purchased from American Type Culture Collection (ATCC number CRL-1442). Cell line was accompanied by identification test certificate and was grown in McCoy's 5a medium according to corresponding tissue culture collection protocols.

### Cell Culture Reagents

Fetal calf serum (FCS) and Dulbecco's minimal essential medium (DMEM) were from Bioconcept (Allschwil, Switzerland). All other cell culture reagents (Trypsin EDTA, Kanamycin, stable Glutamine) were from BioWest (France). All cell culture experiments were performed in TPP (Trasalingen, Switzerland) plastic ware.

### Single Cell Cloning by Serial Dilution

G401 cells were cloned by placing 1 cell/well. The cells were expanded and analyzed by cytogenetic approach. Selected clones were subcloned second time by placing 0.5 cells/well, expanded and their karyograms were analyzed as it was previously described (19). Clones were cultivated until passage 33 and every third passage was analyzed.

### Cytogenetic Studies

Colcemid was from Gibco Life Technologies. Methanol, glacial acetic acid, Trypan blue solution were from Sigma. Cells were incubated with colcemid and metaphases for chromosome spread were observed under phase contrast microscope as it was previously described (19). At least 50 metaphases were analyzed. For image acquisition and analysis of chromosomal bands (karyotyping) the proprietary software (e.g., Genikon) was used.

### Fluorescence *in-situ* Hybridization (FISH)

FISH probes were from Kreatech/Leica. All chemicals were from Sigma. Slides were counterstained with DAPI and observed under fluorescence microscope as it was previously described (19).

### Proliferation Assay

Ten thousand cells per well of cloned G-401 cells were placed into 24-well plates in triplicates in 1 ml of medium. Cells were incubated at 37°C, 5% CO_2_ and cell count was performed on days 3, 5, 7, and 10 as it was previously described (20).

### Cell Cycle Analysis by Flow Cytometry

Cells were stained with propidium iodide (PI) (Thermo Fisher, USA) and analyzed using a FACSAria SORP cell sorter (Becton Dickinson, USA) at Ex.561 nm/Em.575–590 nm for cell cycle distribution as previously described (21). Each measurement was performed in triplicate.

### Fluorescence Microscopy for Analysis of Mitotic Cells

Twenty thousand cells were cultivated on 12 mm cover glasses o/n, fixed with Methanol/Acetone (1:1) and stained with DAPI for 20 min at RT as it was described in Tarnowski et al. (22). Images were collected on Olympus BX-51 microscope with 40X objective and analyzed using proprietary software. At least 300 nuclei were analyzed and for calculation of the percentage of mitotic cells the number of mitotic cells was divided to a total number of investigated nuclei from the same slide × 100%.

### Comparative Genome Hybridization (CGH) -Array

DNA was extracted using DNeasy kit (QIAGEN). CGH-Array was performed with 4 × 180K array (CGX-HD from Perkin Elmer) according to manufacturer's instructions. After hybridization, the array was scanned in a dual-laser scanner (Perkin Elmer) and the images were extracted through CytoGenomics Software (Agilent Technologies) with 37K filter (Backbone resolution 400 kb (oligos every 100 kb) and resolution in the targeted regions 40 kb (oligos every 10 kb). All data were analyzed using Genoglyphix Build Version 3.1-2 (Perkin Elmer).

### Identification of Genes

Genes located in the chromosomal regions of interest were identified and characterized using the Lynx bioinformatics system (23).

## Results

**Figure 1:**
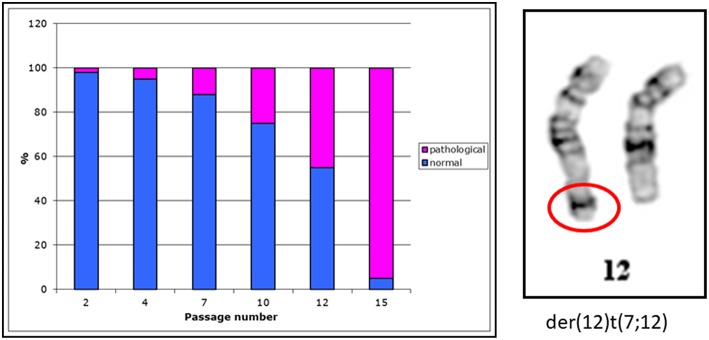
Distribution of normal and pathological metaphases in the G-401 cell line. Metaphases of the G-401 cells. Percentage of normal (46, XY) and pathological (46,XY,der(12)t(7;12) metaphases in the G-401 cell line is shown in a diagram on a left side. With increasing passage number the numbers of metaphases having this derivative chromosome 12 (shown on a right side) were higher, *n* = 50 per passage.

Seventy percent of cancer types diagnosed in children and young adults under 20 years are rare cancers. We have investigated rhabdoid tumor cell line G-401. This cell line was established from a 3-month old infant. In this cell line we have observed cells with an aberrant chromosome 12 (Figure 1, right side). With increasing passage numbers more cells with a derivative chromosome 12 have been observed (Figure 1, left side)

**Figure 2:**
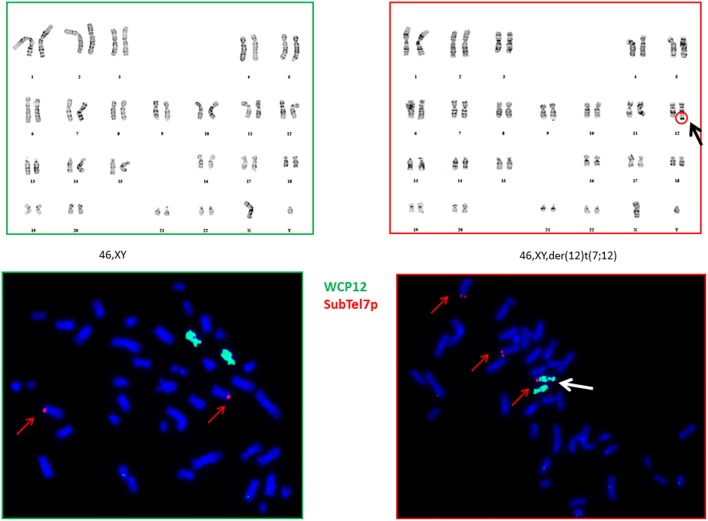
Karyograms and FISH analysis of 2E8 and 3D5 clones. Karyograms: Chromosomal analysis (karyotyping) of G-401 cell line showed a normal karyotype in clone 2E8 (46,XY) on left side and a derivative chromosome 12 as sole anomaly in metaphases of clone 3D5 on right side (46,XY,der(12)t(7;12)(p?14;q?24) **(upper part)**. FISH data: FISH analysis of clones with a normal and aberrant karyotype (2E8 on left side and 3D5 on right side) with commercially available probes WC (Whole Chromosome) 12 (green) and Sub-Telomere 7p (red) revealed an additional 7p segment at the telomeric site of q-arm of one of chromosome 12 (partial trisomy 7p), inducated with a white arrow **(lower part)**.

We have subcloned the G-401 using limiting dilutions and successfully separated cells carrying derivative chromosome 12 from the cells having a normal karyotype. On Figure 2 are shown the karyogram of the 2E8 clone (46, XY) and the karyogram of the 3D5 clone with a 7p translocation (upper part). The FISH analysis showed that these cells carried an additional 7p-segment at the end of the long arm of chromosome 12q (partial trisomy 7p) (Figure 2, lower part). In all analyzed cells both chromosomes 7 were apparently normal.

After second subcloning every third passage has been karyotyped, in total 33 passages have been analyzed. All clones kept their cytogenetic features.

**Figure 3:**
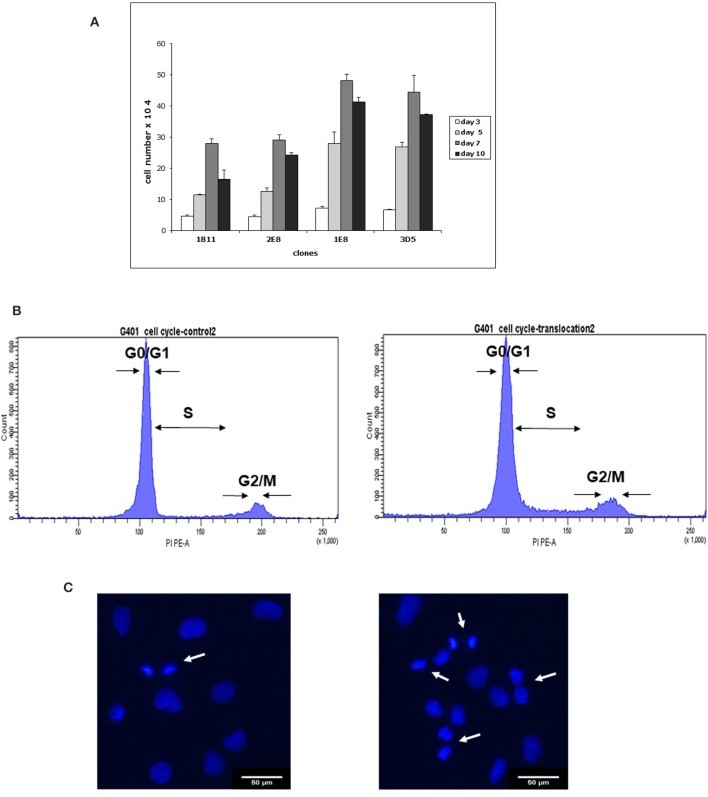
Differences between 2E8 and 3D5 clones in proliferation **(A)**, cell cycle **(B)**, and DAPI staining of the nuclei **(C)**. **(A)** Proliferation assay: The G-401 cell line has been cloned by limiting dilutions and the cells with partial trisomy 7p were separated from the cells having a normal karyotype. Two clones with normal chromosomes and 2 clones with partial trisomy 7p were used in proliferation experiments. Clones with the partial trisomy 7p (1E8 and 3D5) grew more rapidly than the clones with normal chromosomes (1B11 and 2E8). Cell counts were performed on days 3 (white bars), 5 (light gray bars), 7 (dark gray bars), and day 10 (black bars). The percentage of dead cells in cultures did not exceed 10%. Values are cell number means ± SEM of three independent sets of experiments with *n* = 9. **(B)** Cell cycle analysis: Asynchronously dividing cells were stained with PI and measured on FACSAria. Linear gates were set on PI fluorescence distribution histogram to mark G0/G1, S, and G2/M phases of the cell cycle. Cell frequencies in each phase are provided as mean ± SD (*n* = 3). Representative histograms for 2E8 and 3D5 clones are shown. In 2E8 clone (left side) the G0/G1 comprised 85.33 ± 1.31%, S comprised 6.6 ± 1.49% and G2/M comprised 5.9 ± 0.82% cells. In 3D5 clone (right side) G0/G1 phase comprised 75.33 ± 4.25%, S comprised 14.97 ± 0.93%, and G2/M comprised 8.03 ± 3.76%. **(C)** DAPI staining for identification of mitotic cells: Asynchronous cultures of 2E8 and 3D5 cells were used for DAPI staining of the nuclei. Mitotic cells are indicated with an arrow. Scale bar 50 μM. 2E8 cells (46, XY) (on left side) and 3D5 cells (46,XY,der(12)t(7;12) (on right side) were digitally imaged on an Olympus microscope. Mitotic cells are recognized by the bright DAPI staining of their condensed chromatin.

The cell proliferation assay showed that clones possessing the partial trisomy 7p (derivative chromosome 12) grew more rapidly than clones with apparently normal chromosomes (Figure 3A).

On days 3, 7, and 10 there were 1.6 times more cells in clones with a translocation 7p (1E8 and 3 D5) compared with clones having normal karyotype and on day 5 there were almost 2 times more cells in clones with an aberrant chromosome (Figure 3A).

The DNA profiles of these cells were otherwise identical to those of normal karyotype, therefore excluding contamination by other cell cultures (data are not shown).

In cell cycle experiments we stained both 2E8 and 3D5 cells with propidium iodide and analyzed cells on FACSAria. Here we observed a trend for dividing cell prevalence in 3D5 clone compared to 2E8 clone (Figure 3B). In 2E8 cells having normal karyotype the G0/G1, S and G2/M phases comprised 85,33 ± 1,31, 6.6 ± 1,49, and 5.9 ± 0,82%, while in 3D5 cells the G0/G1, S, and G2/M phases comprised 75,33 ± 4,25, 14,97 ± 0,93, and 8,03 ± 3,76%, respectively.

We also performed a DAPI staining of 2E8 and 3D5 clones and counted mitotic cells under fluorescent microscope. In cover glasses with 2E8 cells we observed approximately 20.35 ± 3.83% mitotic nuclei and in 3D5 clone 1,7 times more mitotic nuclei (34,45 ± 2.34%). Figure 3C demonstrates DAPI stained nuclei in both 2E8 and 3D5 clones. Interestingly, it was not possible to perform these experiments on synchronized cells: after 30 h of starvation almost all 2E8 cells died, whereas the clone 3D5 with a 7p translocation continued to do well. For this reason all cell cycle (*n* = 3) and DAPI staining experiments (*n* = 3) were performed with asynchronized cells.

**Figure 4:**
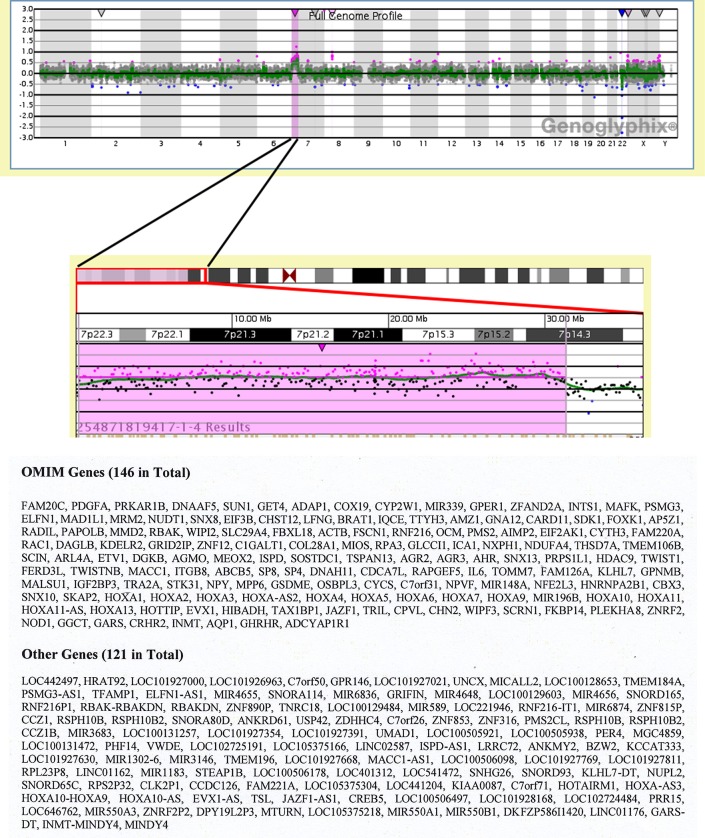
Results of Comparative Genom Hybridization (CGH) array. Full genome profile from Genoglyphix is shown. Terminal gain of chromosome 7p22.3p14.3 Array Result: arr[GRCh37/hg19] 7p22.3p14.3(187243_31346787)x3 **(upper part)**. Genes located in the region of interest are listed: 146 Online Mendelian Inheritance in Man (OMIM) genes and 121 other genes **(lower part)**.

The CGH-Array was performed to characterize more precisely the breakpoint of chromosome rearrangement of derivative chromosome 12. The results have shown that partial trisomy 7 expanded from the band 7p22.3 until 7p14.3 (Figure 4, upper part). We have also listed OMIM (Online Mendelian Inheritance in Man) genes and other genes in this region (Figure 4, lower part).

Functional characterization of the genes in the regions of interest using bioinformatics approach identified a number of genes involved in proliferation (see Supplementary Table 1).

Since the clones with derivative chromosome 12 have three copies of the 7p-region we propose that the presence of third gene alleles of 7p could have provided growth advantage or that genes located in this locus could regulate the activation of other genes and promote proliferation. These genes are a potential target for application of anti-proliferative drugs.

## Discussion

Human solid tumor growth and progression is enabled by the aberrant gene function that positively and negatively regulates cell proliferation, angiogenesis, migration and invasion. Chromosomal heterogeneity is observed in many tumor cell lines. It is understood that chromosomal abnormalities can confer a selective advantage. It is recommended to evaluate during extensive cell passaging the karyotypic profile as a laboratory test control. Currently, microarray based techniques including comparative genomic hybridization and single nucleotide polymorphism analysis are widely used to monitor chromosomal instability. However, classical cytogenetic analysis is still the gold standard for many routine laboratories.

Here we describe chromosomal aberrations in the G-401 cell line. In this rhabdoid cell line (formerly classified as Wilms tumor) we have observed and characterized metaphases with derivative chromosome 12 arising from partial trisomy 7p. With increasing passage number the numbers of metaphases having this derivative chromosome 12 were found to be higher. We were able to subclone G-401 cells by limiting dilutions at passage 12 and successfully separated cells having normal karyotypes from cells having partial trisomy 7p (derivative chromosome 12). Notably cells possessing partial trisomy 7p grew more rapidly compared to the normal karyotype. These findings were demonstrated using proliferation assay, cell cycle experiments and with DAPI staining.

Standard chromosomal investigations alone or combined with other techniques such as fluorescent *in-situ* hybridization (FISH) can be used to confirm observational chromosomal aberrations and can be applied to investigate any tumor cell line particularly applicable for possibly heterogeneous primary cell populations.

Chromosomal aberrations in chromosome 7 are often observed in brain, blood and many other tumors (24–26). An unbalanced der(12)t(7;12) translocation was described in a patient with childhood T-cell acute lymphoblastic leukemia (27). It was speculated that this translocation may be involved in leukemogenesis, but further investigations were not possible because this patient died with the second relapse.

In present study we describe for the first time a 7p triplication in a rare rhabdoid tumor of kidney. It was a sole anomaly in metaphases of the G-401 cell line. Interestingly, both chromosomes 7 are structurally normal. It is unclear when and how this translocation appeared on one of the chromosomes 12 in this cell line. This (der12)t(7;12) translocation is stable, we passaged the clones for a long time (over 33 passages) and all investigated clones kept this 7p translocation. Both clones with a normal karyotype (1B11 and 2E8) did not show any chromosomal abnormalities over 33 passages and had a normal 46, XY karyotype.

Since the clones with partial trisomy 7p have three copies of the 7p-region we propose that the presence of third gene alleles of 7p could have provided the growth advantage or that the genes located in this locus could regulate the activation of other genes and promote the proliferation. In Supplementary Table 1 (which contains information about Gene symbol, Gene description, Refseq summary and Cancer gene index) are listed the genes which may contribute to a faster proliferation of clones with 7p triplication. Among these genes are Il-6, PDGFA, ETV-1, and RAC-1.

Il-6 is a pleiotropic cytokine that plays an important role in inflammation and the maturation of B-cells. It induces proliferation, differentiation and dedifferentiation and acts on many cells (28, 29).

PDGFA protein is involved in a number of biological processes, including hyperplasia, embryonic neuron development. It is a strong mitogen for a variety of cell types, e.g., connective tissue, smooth muscle cells, and bone cells (30–32).

ETV1 protein encoded by *ETV* gene is a member of the ETS (E twenty-six) family of transcription factors. The ETS proteins regulate many target genes that modulate biological processes like cell growth, angiogenesis, migration, proliferation and differentiation (33, 34).

RAC1 protein encoded by *RAC*1 gene regulates many cellular events including the control of cell growth, cytoskeletal reorganization, and the activation of protein kinases (35, 36).

The clones with normal chromosomes and clones with partial trisomy 7p described in this study could be used as a preclinical model to study the involvement of partial chromosome 7 alterations in the development of rhabdoid tumors. These models might be also used for evaluating of anti-cancer drugs in preclinical studies.

## Data Availability

The datasets generated for this study are available on request to the corresponding author.

## Author Contributions

EF-K, NA, and SR performed cytogenetic experiments. DC, F-MH, MM, and AB performed cloning and proliferation experiments. NM collected gene expression data and analyzed them as well as prepared the Supplementary Material. SB, MM, and NA contributed to the study design, concept development, and manuscript writing. EF-K designed the whole study, participated in data collection and analysis, and wrote a manuscript.

### Conflict of Interest Statement

EF-K and SB have received the Batzebär grant. The remaining authors declare that the research was conducted in the absence of any commercial or financial relationships that could be construed as a potential conflict of interest. The handling editor declared a shared affiliation, though no other collaboration, with several of the authors EF-K and F-MH.
