# Supplementary material for: Chromosomal Heterogeneity of the G-401 Rhabdoid Tumor Cell Line: Unusual Partial 7p Trisomy
Source: Front Med (Lausanne). 2019 Aug 30;6:187. doi: 10.3389/fmed.2019.00187 (PMC6729120; doi:10.3389/fmed.2019.00187)
Supplement: Supplementary file 1 [file Table_1.DOCX]

**Chromosomal heterogeneity of the G-401 rhabdoid tumor cell line: unusual partial 7p trisomy**

Elizaveta Fasler-Kan^1,2*^, Nijas Aliu^3*^, Frank-Martin Haecker^4,5^, Natalia Maltsev^6^, Sabrina Ruggiero^1^, Dietmar Cholewa^1^, Andreas Bartenstein^1^, Milan Milošević^1^ and Steffen M. Berger^1^

*^1^Department. of Pediatric Surgery, Children’s Hospital, Inselspital, University of Bern and Department of Biomedical Research, University of Bern, Switzerland*

*^2^Department of Biomedicine, University of Basel und University Hospital Basel, Switzerland*

*^3^Department of Human Genetics, University Children’s Hospital, Inselspital, Bern, Switzerland*

*^4^Department of Pediatric Surgery, Children’s Hospital of Eastern Switzerland, St. Gallen, Switzerland*

*^5^Faculty of Medicine, University of Basel, Basel, Switzerland*

*^6^Department of Human Genetics and USA Computation Institute, University of Chicago, Chicago, USA.*

Supplementary Material Table 1.

| Gene Symbol | Gene  Description | Refseq Summary | Cancer Gene  Index (CGI) |
| --- | --- | --- | --- |
| IL6  3569 | interleukin 6 | This gene encodes a cytokine that functions in inflammation and the maturation of B cells. In addition, the encoded protein has been shown to be an endogenous pyrogen capable of inducing fever in people with autoimmune diseases or infections. The protein is primarily produced at sites of acute and chronic inflammation, where it is secreted into the serum and induces a transcriptional inflammatory response through interleukin 6 receptor, alpha. The functioning of this gene is implicated in a wide variety of inflammation-associated disease states, including suspectibility to diabetes mellitus and systemic juvenile rheumatoid arthritis. Alternative splicing results in multiple transcript variants. [provided by RefSeq, Dec 2015]. |  |
| HOXA7  3204 | homeobox A7 | In vertebrates, the genes encoding the class of transcription factors called homeobox genes are found in clusters named A,, C, and D on four separate chromosomes. Expression of these proteins is spatially and temporally regulated during embryonic development. This gene is part of the A cluster on chromosome 7 and encodes a DNA-binding transcription factor which may regulate gene expression, morphogenesis, and differentiation. For example, the encoded protein represses the transcription of differentiation-specific genes during keratinocyte proliferation, but this repression is then overcome by differentiation signals. This gene is highly similar to the antennapedia (Antp) gene of Drosophila. [provided by RefSeq, Jul 2008]. |  |
| GGCT  79017 | gamma-glutamyl  cyclotransferase | The protein encoded by this gene catalyzes the formation of 5-oxoproline from gamma-glutamyl dipeptides, the penultimate step in glutathione catabolism, and may play a critical role in glutathione homeostasis. The encoded protein may also play a role in cell proliferation, and the expression of this gene is a potential marker for cancer. Pseudogenes of this gene are located on the long arm of chromosome 5 and the short arm of chromosomes 2 and 20. Alternatively spliced transcript variants encoding multiple isoforms have been observed for this gene. [provided by RefSeq, Dec 2010]. |  |
| CHN2  1124 | chimerin 2 | This gene encodes a guanosine triphosphate (GTP)-metabolizing protein that contains a phorbol-ester/diacylglycerol (DAG)-type zinc finger, a Rho-GAP domain, and an SH2 domain. The encoded protein translocates from the cytosol to the Golgi apparatus membrane upon binding by diacylglycerol (DAG). Activity of this protein is important in cell proliferation and migration, and expression changes in this gene have been detected in cancers. A mutation in this gene has also been associated with schizophrenia in men. Alternative transcript splicing and the use of alternative promoters results in multiple transcript variants. [provided by RefSeq, May 2014]. |  |
| ETV1  2115 | ETS variant 1 | This gene encodes a member of the ETS (E twenty-six) family of transcription factors. The ETS proteins regulate many target genes that modulate biological processes like cell growth, angiogenesis, migration, proliferation and differentiation. All ETS proteins contain an ETS DNA-binding domain that binds to DNA sequences containing the consensus 5'-CGGA[AT]-3'. The protein encoded by this gene contains a conserved short acidic transactivation domain (TAD) in the N-terminal region, in addition to the ETS DNA-binding domain in the C-terminal region. This gene is involved in chromosomal translocations, which result in multiple fusion proteins including EWS-ETV1 in Ewing sarcoma and at least 10 ETV1 partners (see PMID: 19657377, Table 1) in prostate cancer. In addition to chromosomal rearrangement, this gene is overexpressed in prostate cancer, melanoma and gastrointestinal stromal tumor. Multiple alternatively spliced transcript variants encoding different isoforms have been identified. [provided by RefSeq, Jul 2016]. |  |
| ITGB8  3696 | integrin subunit  beta 8 | This gene is a member of the integrin beta chain family and encodes a single-pass type I membrane protein with a VWFA domain and four cysteine-rich repeats. This protein noncovalently binds to an alpha subunit to form a heterodimeric integrin complex. In general, integrin complexes mediate cell-cell and cell-extracellular matrix interactions and this complex plays a role in human airway epithelial proliferation. Alternatively spliced variants which encode different protein isoforms have been described; however, not all variants have been fully characterized. [provided by RefSeq, Jul 2008]. |  |
| SOSTDC1  25928 | sclerostin  domain  containing 1 | This gene is a member of the sclerostin family and encodes an N-glycosylated, secreted protein with a C-terminal cystine knot-like domain. This protein functions as a bone morphogenetic protein (BMP) antagonist. Specifically, it directly associates with BMPs, prohibiting them from binding their receptors, thereby regulating BMP signaling during cellular proliferation, differentiation, and programmed cell death. [provided by RefSeq, Jul 2008]. |  |
| AHR  196 | aryl hydrocarbon  receptor | The protein encoded by this gene is a ligand-activated helix-loop-helix transcription factor involved in the regulation of biological responses to planar aromatic hydrocarbons. This receptor has been shown to regulate xenobiotic-metabolizing enzymes such as cytochrome P450. Before ligand binding, the encoded protein is sequestered in the cytoplasm; upon ligand binding, this protein moves to the nucleus and stimulates transcription of target genes. [provided by RefSeq, Sep 2015]. | c6 glioma |
| PDGFA  5154 | platelet derived  growth factor  subunit A | This gene encodes a member of the protein family comprised of both platelet-derived growth factors (PDGF) and vascular endothelial growth factors (VEGF). The encoded preproprotein is proteolytically processed to generate platelet-derived growth factor subunit A, which can homodimerize, or alternatively, heterodimerize with the related platelet-derived growth factor subunit B. These proteins bind and activate PDGF receptor tyrosine kinases, which play a role in a wide range of developmental processes. Alternative splicing results in multiple transcript variants. [provided by RefSeq, Oct 2015]. | glioma,  high-grade  glioma |
| FSCN1  6624 | fascin  actin-bundling  protein 1 | This gene encodes a member of the fascin family of actin-binding proteins. Fascin proteins organize F-actin into parallel bundles, and are required for the formation of actin-based cellular protrusions. The encoded protein plays a critical role in cell migration, motility, adhesion and cellular interactions. Expression of this gene is known to be regulated by several microRNAs, and overexpression of this gene may play a role in the metastasis of multiple types of cancer by increasing cell motility. Expression of this gene is also a marker for Reed-Sternberg cells in Hodgkin's lymphoma. A pseudogene of this gene is located on the long arm of chromosome 15. [provided by RefSeq, Sep 2011]. | glioma, glioblastomas, |
| NPY  4852 | neuropeptide Y | This gene encodes a neuropeptide that is widely expressed in the central nervous system and influences many physiological processes, including cortical excitability, stress response, food intake, circadian rhythms, and cardiovascular function. The neuropeptide functions through G protein-coupled receptors to inhibit adenylyl cyclase, activate mitogen-activated protein kinase (MAPK), regulate intracellular calcium levels, and activate potassium channels. A polymorphism in this gene resulting in a change of leucine 7 to proline in the signal peptide is associated with elevated cholesterol levels, higher alcohol consumption, and may be a risk factor for various metabolic and cardiovascular diseases. The protein also exhibits antimicrobial activity against bacteria and fungi. [provided by RefSeq, Oct 2014]. | c6 glioma,  metastatic neuroblastoma |
| MIR148A  406940 | microRNA 148a |  |  |
| HNRNPA2B1  3181 | heterogeneous  nuclear  ribonucleoprotein  A2/B1 | This gene belongs to the A/B subfamily of ubiquitously expressed heterogeneous nuclear ribonucleoproteins (hnRNPs). The hnRNPs are RNA binding proteins and they complex with heterogeneous nuclear RNA (hnRNA). These proteins are associated with pre-mRNAs in the nucleus and appear to influence pre-mRNA processing and other aspects of mRNA metabolism and transport. While all of the hnRNPs are present in the nucleus, some seem to shuttle between the nucleus and the cytoplasm. The hnRNP proteins have distinct nucleic acid binding properties. The protein encoded by this gene has two repeats of quasi-RRM domains that bind to RNAs. This gene has been described to generate two alternatively spliced transcript variants which encode different isoforms. [provided by RefSeq, Jul 2008]. |  |
| TWIST1  7291 | twist family bHLH transcription factor 1 | This gene encodes a basic helix-loop-helix (bHLH) transcription factor that plays an important role in embryonic development. The encoded protein forms both homodimers and heterodimers that bind to DNA E box sequences and regulate the transcription of genes involved in cranial suture closure during skull development. This protein may also regulate neural tube closure, limb development and brown fat metabolism. This gene is hypermethylated and overexpressed in multiple human cancers, and the encoded protein promotes tumor cell invasion and metastasis. Mutations in this gene cause Saethre-Chotzen syndrome in human patients, which is characterized by craniosynostosis, ptosis and hypertelorism. [provided by RefSeq, Aug 2017]. |  |
| RAC1  5879 | Rac family small GTPase 1 | The protein encoded by this gene is a GTPase which belongs to the RAS superfamily of small GTP-binding proteins. Members of this superfamily appear to regulate a diverse array of cellular events, including the control of cell growth, cytoskeletal reorganization, and the activation of protein kinases. Two transcript variants encoding different isoforms have been found for this gene. [provided by RefSeq, Mar 2009]. | glioma,  glioblastoma, glioblastoma multiforme |
| PMS2  5395 | PMS1 homolog 2, mismatch repair system component | The protein encoded by this gene is a key component of the mismatch repair system that functions to correct DNA mismatches and small insertions and deletions that can occur during DNA replication and homologous recombination. This protein forms heterodimers with the gene product of the mutL homolog 1 (MLH1) gene to form the MutL-alpha heterodimer. The MutL-alpha heterodimer possesses an endonucleolytic activity that is activated following recognition of mismatches and insertion/deletion loops by the MutS-alpha and MutS-beta heterodimers, and is necessary for removal of the mismatched DNA. There is a DQHA(X)2E(X)4E motif found at the C-terminus of the protein encoded by this gene that forms part of the active site of the nuclease. Mutations in this gene have been associated with hereditary nonpolyposis colorectal cancer (HNPCC; also known as Lynch syndrome) and Turcot syndrome. [provided by RefSeq, Apr 2016]. | glioblastoma |
| GPNMB  10457 | glycoprotein nmb | The protein encoded by this gene is a type I transmembrane glycoprotein which shows homology to the pMEL17 precursor, a melanocyte-specific protein. GPNMB shows expression in the lowly metastatic human melanoma cell lines and xenografts but does not show expression in the highly metastatic cell lines. GPNMB may be involved in growth delay and reduction of metastatic potential. Two transcript variants encoding different isoforms have been found for this gene. [provided by RefSeq, Jul 2008]. | glioblastoma multiforme,  glioma,  malignant  gliomas, |
| AQP1  358 | aquaporin 1 (Colton blood group) | This gene encodes a small integral membrane protein with six bilayer spanning domains that functions as a water channel protein. This protein permits passive transport of water along an osmotic gradient. This gene is a possible candidate for disorders involving imbalance in ocular fluid movement. [provided by RefSeq, Aug 2016]. | glioblastomas |
| ACTB  60 | actin beta | This gene encodes one of six different actin proteins. Actins are highly conserved proteins that are involved in cell motility, structure, integrity, and intercellular signaling. The encoded protein is a major constituent of the contractile apparatus and one of the two nonmuscle cytoskeletal actins that are ubiquitously expressed. Mutations in this gene cause Baraitser-Winter syndrome 1, which is characterized by intellectual disability with a distinctive facial appearance in human patients. Numerous pseudogenes of this gene have been identified throughout the human genome. [provided by RefSeq, Aug 2017]. | glioma |
| PRKAR1B  5575 | protein kinase cAMP-dependent type I regulatory subunit beta | The protein encoded by this gene is a regulatory subunit of cyclic AMP-dependent protein kinase A (PKA), which is involved in the signaling pathway of the second messenger cAMP. Two regulatory and two catalytic subunits form the PKA holoenzyme, disbands after cAMP binding. The holoenzyme is involved in many cellular events, including ion transport, metabolism, and transcription. Several transcript variants encoding the same protein have been found for this gene. [provided by RefSeq, Aug 2015]. |  |
